# Shortage of plasma-derived medicinal products: what is next? narrative literature review on its causes and counteracting policies in Italy

**DOI:** 10.3389/fphar.2024.1375891

**Published:** 2024-05-06

**Authors:** Matteo Bolcato, Claudio Jommi

**Affiliations:** ^1^ Department of Neuroscience, University of Padova, Padova, Italy; ^2^ Department of Pharmaceutical Sciences, Università del Piemonte Orientale, Novara, Italy

**Keywords:** plasma-derived medicinal products, market, supply, demand, policy, shortage

## Abstract

**Introduction:** This paper describes the peculiarities of the plasma-derived medicinal product (PDMP) market and illustrates the results of a review of the literature on policies aimed at counteracting the shortage of PDMPs.

**Characteristics of PDMPs:** Plasma is primarily used for the industrial production of blood products (80%). The demand for PDMPs, particularly immunoglobulins (IGs), is increasing. However, the production of PDMPs is complex, long (7**–**12 months), and expensive, accounting, according to US estimates, for 57% of the total costs of PDMPs compared to 14% for small molecules.

**PDMP market:** Unexpected increases in clinical need cannot be addressed in the short term. Once the demand for some diseases is satisfied, the collection and fractionation of plasma will only be used to supply some specific patients. Hence, the full weight of the marginal costs, which remain constant, are borne by a few products. According to last liter economics, the industry stops producing when the marginal revenue equals the marginal cost, thereby reducing the convenience of producing the most commonly used PDMPs (albumin and IG). The imbalance between the demand and supply of PDMPs was exacerbated by the COVID-19 pandemic, which further increased the cost of plasma collection.

**Shortage issue and possible solutions:** Policies to counteract this imbalance have also been discussed. If the demand is inappropriate, it should be reduced. If the demand is appropriate and supply cannot be increased, the demand should be prioritized for patients for whom PDMPs are the only available treatment. If the shortage depends on insufficient supply and technical and allocative efficiency, both production and supply should be improved, together with incentives for all stakeholders involved in the PDMP market to increase the sustainability of production/supply. The paper is focused on this second issue, that is supply-driven unbalance.

## Introduction

This study explores the characteristics of the plasma-derived medicinal product (PDMP) market, reasons for the shortage of PDMPs, and policies aimed at addressing shortages. We conducted a literature review using Embase, which is more appropriate for economic topics related to health and healthcare than other databases. The key words used referred in general to PDMPs (“plasma-derive*” “plasma derived medicinal product” “supply*” “demand*” “industr*” “market*”, “patient blood management”, with exclusion of “clinical study”) and more specifically to immunoglobulins (IG), that were more affected by market shortages (“immunoglobulin” “self-sufficienc*” “shortage*” “supply*” “demand*” “industr*” “market*”). Papers were retrieved for 2000–2023 years. Peer-reviewed literature was complemented by papers and documents retrieved from the gray literature.

The subsequent sections of the study are organized as follows. The first section presents the characteristics of PDMPs and their main indications. The second discusses the demand, supply, regulation, and self-sufficiency of the PDMP market and its differences from other medicines. The third section highlights the shortcomings and possible solutions to the existing challenges.

### Plasma-derived medicinal products: characteristics and therapeutic indications

PDMPs are produced using industrial plasma manufacturing processes. Plasma is a blood component originating from the voluntary donation of biological material by donors, which is conducted according to two models: a) the public model, for example, in Italy, where the donation is voluntary and unpaid, or b) the private model, such as in the United States, where the donation is voluntary and paid.

Plasma is not only used for the industrial production of blood products but also in the healthcare sector as a blood component, although its clinical indications are becoming increasingly restrictive.

The production of PDMPs is particularly complex, highly standardized, and regulated. Donated plasma is collected either in public transfusion departments or in specialized centers (included those of the pharmaceutical companies) with expertise in blood separation and plasmapheresis (a technique used to separate plasma from the remaining blood components that are immediately re-infused into the original donor). The production process comprises several phases. The first is fractionation, which is a chemical-physical process aimed at separating plasma proteins (albumin, immunoglobulins, coagulation factors, and other proteins of interest in some rare diseases). The following production phases remove and inactivate potentially dangerous microorganisms, involving purification, viral inactivation/removal, and aseptic filling or freeze-drying. At the end of these processes and controls, the drug is considered safe for packaging and subsequent distribution to the patients. Thereafter, the safety is constantly monitored through pharmacovigilance.

PDMPs are indicated for the treatment of several severe diseases and, in some cases, in particular as regards as immunoglobulins (IGs), cannot be replaced by other products for the treatment of acute and chronic clinical conditions.

The strategic and pivotal role of PDMPs has been confirmed by the inclusion of IGs and coagulation factors (VIII and IX) in the list of drugs considered essential for basic healthcare services by the World Health Organization (World Health Organization, 2021).

The most commonly used PDMPs in clinical settings and the international market are as follows:

➢ Albumin—used for treating liver cirrhosis and its complications, aimed at increasing the general oncotic pressure. It is also necessary to treat hypovolemic and septic shock, burns, severe neonatal jaundice, and nephrotic or malabsorption syndromes.
➢ Coagulation factors (VII, VIII, IX, X, and von Willebrand) are required to treat coagulation diseases and disorders, such as hemophilia A and B, von Willebrand disease, and clotting factor deficiency due to liver diseases.
➢ Specific IGs are necessary to prevent the development of hepatitis B, tetanus, cytomegalovirus infections, maternal-fetal Rh factor incompatibility, and immunomodulation in organ transplants.
➢ Polyvalent immunoglobulins are necessary for prophylaxis against infections in patients with primary or secondary immunodeficiencies and in certain neurological pathologies (chronic inflammatory demyelinating polyradiculoneuropathy, multifocal motor neuropathy, or Guillain–Barré syndrome).
➢ Antithrombin III, a drug used to treat congenital or acquired antithrombin III deficiencies.
➢ Prothrombin complex concentrate is used for the treatment and prophylaxis of bleeding episodes.
➢ Fibrinogen, a drug used for the treatment of congenital or acquired deficiency of this specific factor and hemorrhagic disorders.
➢ Alpha1-proteinase inhibitor, a drug used to treat alpha1-antitrypsin deficiency.
➢ C1-esterase inhibitor, which is used to treat the clinical manifestations of hereditary angioedema.

The use of IGs deserves particular attention (Prevot and Jolles, 2020). As discussed below, there has been a consistent increase in their use.

IGs are available as sterile preparations of concentrated antibodies extracted from the plasma for subcutaneous or intravenous administration.

The primary established indications for intravenously administered IGs are as follows (Kivity et al., 2010; Perez et al., 2017):

➢ Primary immunodeficiency syndromes with impaired antibody production.
➢ Secondary immunodeficiencies in patients with severe or recurrent infections, ineffective antimicrobial treatment, and inability to produce specific antibodies.
➢ Immunomodulation in adults, children and adolescents for:
   o Primary Immune Thrombocytopenia in patients at high risk of bleeding or before surgery to restore platelet count.
   o Guillain–Barré syndrome.
   o Kawasaki disease (in conjunction with acetylsalicylic acid).
   o Chronic inflammatory demyelinating polyneuropathy.
   o Multifocal motor neuropathy.

Intravenously administered immunoglobulins are also commonly used drugs for the following clinical indications:

➢ Myasthenic crisis.
➢ Rapid worsening forms of myasthenia gravis and disease exacerbation when a rapid improvement in muscle strength is required to minimize the risk of bulbar palsy or respiratory failure.
➢ In the initial stages of myasthenia gravis, when the effects of cortisone and/or immunosuppressive therapy are anticipated.
➢ As preparation for thymectomy, in patients with myasthenia gravis.
➢ Patients with myasthenia gravis who are unresponsive to steroid and/or immunosuppressive drug therapies or have contraindications to their use.

Subcutaneously administered immunoglobulins are licensed for the following:

➢ Primary immunodeficiency syndromes with impaired antibody production.
➢ Hypogammaglobulinemia and recurrent bacterial infections in patients with chronic lymphocytic leukemia, who do not respond to antibiotic prophylaxis or in whom antibiotic prophylaxis is contraindicated.
➢ Hypogammaglobulinemia and recurrent bacterial infections in patients with multiple myeloma.
➢ Hypogammaglobulinemia in patients undergoing allogeneic hematopoietic stem cell transplantation.

Administration of IGs is considered life-saving in many of these pathological conditions. The medical need for IGs has grown rapidly due to several factors (Kerr et al., 2014; Farrugia, 2021; Hogan Lovells, 2022; Quinn et al., 2022): a) general aging of the population, which is potentially more in need of treatment and medicines; b) patients with an indication for the administration of IGs who, thanks to the effectiveness of this treatment, have a longer life expectancy, thus making a correspondingly longer therapy need; c) increased diagnosis rates of diseases that can benefit from the administration of IGs thanks to scientific progress and the possibility of carrying out more accurate (even genetic) diagnoses; d) identification of new therapeutic indications for IGs as a common side effect of new treatments, such as immunotherapy in oncology, resulting in secondary immunodeficiency and immune-mediated neurological and rheumatological diseases.

### Market dynamics: PDMPs vs. other medicines

Market dynamics depend on the demand (consumers) and supply (producers). The demand for any product (good or service) is affected by the need for and perceived value of the product compared to existing alternatives, relative price, and available budget. Producers are generally driven by profit maximization, that is, by maximizing the difference between revenue and cost. Free interaction between demand and supply enables the efficient allocation of resources, optimizing the satisfaction of needs with the available resources if the demand and supply are separated from each other and fully informed, if the consumers are rational (the price they are able to pay reflects the value of the product), and the suppliers are sufficiently numerous to generate competitive pricing. Otherwise, the market should not be left to the free interaction between demand and supply, but should be regulated. Generally, the healthcare sector is affected by market failures, including monopolies generated by patent protection, limited information, and physicians who simultaneously demand healthcare for their patients and supply healthcare services, with the risk that they are not perfect agents in the way they demand healthcare on behalf of their patients. For this reason, healthcare markets are often regulated. For example, the industry is not free to set prices because it can exploit monopolistic power, imposing a very high economic burden on consumers. Furthermore, market mechanisms are based on price discrimination: demand is affected not only by consumers’ willingness but also by their ability to pay, which depends on budget constraints. Price discrimination is generally not acceptable in the healthcare sector, and third-party payers—namely, the government, social insurance, and private insurance—cover healthcare costs, collecting resources through taxes, payroll taxes, and insurance premiums, respectively.

Market regulation implies that domains that would not have been considered by demand (need, comparative value, and budget constraints) and supply (costs and revenues) are mediated by regulators. The existence of third-party payers may drive demand well beyond the level that would have been reached if patients themselves had to pay. Prioritization, guidelines, and, for some products, copayments (i.e., a partial reintroduction of prices) can be used to govern the demand for healthcare.

On this aspect, PDMPs are similar to other medicines. Patients’ need depends on disease severity, that is, the prognosis (life expectancy), quality of life, and economic burden of the disease. It has been demonstrated, for example, that hemophilia, which is one of the target of PDMPs, generates an average 0.69 utility score, where utility is an indicator of Health-related Quality of Life (HR-QoL) ranging from 0 (death/vegetative state) to one (perfect health), severe hemophilia produces 0.197 Disability Adjusted Life Years, i.e., 20% years lost because of premature death or disability (compared to 12% for diabetes, 16.7% for major depression (Kluszczynski et al., 2020). The economic burden of hemophilia in Europe ranged from €67 k in Spain to €195 k in Germany (Cavazza et al., 2016).

The comparative value depends on the added therapeutic value (impact on survival and HR-QoL and/or endpoint surrogating final outcomes) and other value domains, including patient preferences (e.g., better administration route) and the impact on the healthcare organization (e.g., oral vs. intravenous administration). Price negotiation is generally more focused on added therapeutic value; the larger the added therapeutic value, the higher the premium price with respect to comparators (Vogler et al., 2017). Value domains can be more discretionally included in value-for-money considerations or embedded into specific indicators such as the incremental cost-effectiveness ratio (Jommi et al., 2020). Regardless of the method used to convert the value into an acceptable price, the time horizon of the evaluation and the perspective used to estimate avoidable costs may have an important impact on the value for money. The longer the time horizon and broader the perspective used, the higher the probability of appreciating the full impact of a new medicine.

In their price requests, the industry (supply side) considers revenues and costs. The former depends on prices and volumes; the smaller the expected volumes (e.g., medicines for rare diseases, which are the target for many PDMPs) (Strengers, 2023), the higher the price request. The costs depend on the unit prices and quantity of input used (personnel, consumables, services, equipment, etc.) for research and development (R&D), production, selling, and general and administrative expenses. In this respect, PDMPs share with other medicines the peculiarity of high R&D costs, including joint and sunk costs.

Unlike most other medicines and particular drugs derived from chemical synthesis, PDMPs are characterized, as previously mentioned, by a complex, long, and expensive production process. It has been estimated that the time required to move from plasma donation to treatment administration can be as long as 7–12 months (Farrugia and Scaramuccia, 2017) and the manufacturing costs (including plasma as raw material) account for 57% of the total costs of PDMPs, compared to 14% for small molecules (Grabowski and Manning, 2018). The latter estimates refer to US production costs which are affected by donor fees, but, since US is the largest producer and exporter of PDMPs, they affect the production costs of PDMPs used worldwide in general. Another characteristic of the production process is its rigidity. Due to the long lead times, unexpected increases in clinical need cannot be addressed in the short term, and diverse sources of plasma should be used, as possible shortages in the availability of blood in one country could be compensated for by others.

Another peculiarity of PDMPs is the joint nature of their plasma samples. Fractioned plasma is used for different PDMPs, which are indicated for different diseases with different prevalence. Once the demand for very rare diseases has been satisfied, the collection and fractionation of plasma will serve the production of PDMPs targeting only patients suffering from diseases with a higher prevalence, making the marginal (additional) revenue lower, while the marginal (additional) cost remains constant. The industry will continue to produce until the marginal revenue is higher than the marginal cost of producing 1 L and stops producing when the marginal revenue equals the marginal cost (last liter economics). The last liter economics reduces the convenience of producing PDMPs that are used for larger patient population, such as albumin and IGs (Kluszczynski et al., 2020).

### Plasma and self-sufficiency

The production of PDMP, for which recombinant factor products are not available, depends on plasma collection. The WHO has recommended self-sufficiency in safe blood and blood products based on voluntary non-remunerated donations (WHO Expert Group, 2012), but the achievability of this goal has been questioned (Flanagan, 2014; Farrugia and Scaramuccia, 2017). In fact, few countries have reached self-sufficiency in plasma (Rock et al., 2000) and must rely on an open market. Europe imported 38% of its plasma need for fractionation and is reliant on plasma imports from the US (Haanperä et al., 2021). In 2019, North America (mainly the US) accounted for 67% of plasma fractionation, that is, it is the main source of PDMPs used commercially; 18% of the plasma sourced comes from the Asia-Pacific region, of which 75% from China; the contribution of Europe is 14%, although Europe is the largest supplier of recovered plasma (Strengers, 2023). This scenario makes supply extremely sensitive to increases in plasma collection costs in the US.

On July 2022, a proposal for a regulation on substances of human origin (SoHO) for human application was published by the European Commission. To date, it is in the legislative process and presumably will replace the largely outdated legislation on Blood, Tissue and Cells (BTC legislation). Thanks to this regulation, the European Parliament will put in place a number of positive actions that can help improve plasma availability in Europe (The European Parliament and the Council, 2022).

Italy implemented a plasma self-sufficiency program under Law 219/2005. According to this program, blood is collected through the public system (blood centers of the Italian National Health Service - INHS) and owned by the Regions. PDMPs are produced by the industry on behalf of the INHS, based on agreements with the Regions or Regional networks (Lanzoni et al., 2013). If the PDMPs volume coming from the national blood system is insufficient, the INHS can buy it from the open market. The pharmaceutical companies indeed can collect plasma in countries where its commercial use is allowed to obtain products that can be sold on the open market (after price negotiation with national authorities) (United States, Austria, the Czech Republic, Germany, Hungary, Ukraine, and China) (Strengers, 2023). In 2021, PDMPs produced via a self-sufficiency program in Italy covered 71% of the demand, ranging from 10% for subcutaneous IGs (82% for intravenous IGs) to 100% for Factor VIII (Candura et al., 2023).

### PDMPs’ shortage

The shortage of PDMPs, particularly IGs, is a consequence of higher demand-over-supply. This was also previously observed. For example, in the fall of 1997, a shortage of intravenous immunoglobulin occurred in the US because of the recall of some products, and although the shortage was spontaneously overcome, a more proactive intervention of regulators was advocated for future emergencies (Boulis et al., 2002).

On the one hand, supply issues include risk of deviation and non-excellent planning of consumption. IGs supply difficulties, become even worse during the pandemic period, have negatively affected plasma collection (Hartmann and Klein, 2020; Covington et al., 2022). In the US, plasma collection decreased by 18% in 2020. Worldwide, this decline amounted to 14.5% (Strengers, 2023). In Italy, after a decrease in 2020 (−1.7%), volumes of plasma increased by 2% in 2021. Despite a partial recovery, the 2021 increase was lower than the average growth rate of previous years (Candura et al., 2023), making it more difficult to achieve self-sufficiency. As expected, the decrease in the supply of plasma increases its cost. The cost per liter of plasma has increased by 59.3% in Europe and 40.7% in the United States between 2017 and 2022, moving from €108 to €172 in Europe and from $162 to $228 in the United States (Marketing Research Bureau, 2020). In the last 5 years, donor fees have increased by 30% in the US to better compensate donors and counteract the decrease in plasma collection (Brown, 2020; Bernini, 2021). An increase in the cost of plasma has impacted the sustainability of PDMP production. During the pandemic period, in Italy IGs have been particularly affected by shortages, which have also caused rationing in the case of diseases for which they have been prioritized (AIFA, 2022). Even in countries with a high supply of commercially-sourced plasma (like Germany and the US), shortages of intravenous/subcutaneous IGs have occurred (Strengers, 2023).

The demand for PDMPs has increased over time. This increase is motivated not only by the availability of new products or new indications for existing PDMPs but also by higher dosages to enhance PDMP effectiveness (Kluszczynski and Barbosa Eitel, 2022). This demand is expected to increase in the future, considering the diagnostic gaps that are required to be filled (Strengers, 2023). This increase is particularly significant for IGs globally (Farrugia et al., 2019) and their demand is expected to rise by 30% by 2030 (CSL Behring, 2022; Kluszczynski and Barbosa Eitel, 2022). In Italy, despite efforts to fulfill this demand with the self-sufficiency program (Farrugia et al., 2019), a large proportion is still not covered (36% for IGs in 2021) (Candura et al., 2023).

### The supply/demand gap: what policies should be implemented?

Policies aimed at reducing the imbalance between the supply and demand should address the right target.

An excess of the demand over the supply can be addressed by two possible actions. If the demand is inappropriate, it should be reduced. If the demand is appropriate but the supply cannot be increased, the demand should be prioritized to the highest unmet need; for example, for patients for whom PDMPs are the only available treatment. The two options seem interconnected and the debate on clinical use, and particularly on the evidence supporting off-label use, is still controversial (Farrugia and Poulis, 2001; Brand et al., 2021; Kluszczynski and Barbosa Eitel, 2022; Strengers, 2023).

As a consequence of the pandemic, prioritization programs for IG use have been implemented in Italy (AIFA, 2022) and other Western Countries (ANSM, 2018; Whittington Health NHS, 2018; Canadian Blood Services and National Advisory Committee on Blood and Blood Products, 2020).

The guidance document prepared in Italy by AIFA (Agenzia Italiana del Farmaco - Italian Medicines Agency) and the National Blood Center includes prioritization guidelines for IGs similar to those of the Canadian Blood Services and National Advisory Committee on Blood and Blood Products (Canadian Blood Services and National Advisory Committee on Blood and Blood Products, 2020), and containment guidance for their use in specific therapeutic areas depending on the availability of the products.

Although it appears necessary and useful to minimize the consequences caused by IG shortages and to call for rigor in their administration, this model appears to be a new obligatory model of distributive justice. In fact, it may generate, depending on the situation, different IG prescriptions and usage behaviors for patients in different regions. This extremely complex situation has never occurred in many respects and risks not allowing the full application of the principles on which a public health system is based, as it should ensure universality and equal access for equal health needs. This can lead to social imbalances and medicolegal risks in the event of difficulty in accessing appropriate and necessary care.

The supply shortage can be addressed through three possible actions.

The first is an increase in plasma collection. This is not easy to implement because, the determinants of plasma donation may vary a lot. Major motivations of blood donors, new or regular, appear related to the prosocial nature of donation (i.e., altruism, communitarianism, etc.), to personal values (i.e., moral norms), and to the comfort of the donation environment. It was also recalled the importance of personal gain as a motivation for the first donation (going from personal satisfaction to taking time on their work hours) (Beurel et al., 2017). Furthermore, the current supply of plasma suffers from specific contingencies, including military conflicts (the war in Ukraine and the plasma supply in Ukraine) and newly emerging pathogens (SARS-CoV-2) (Strengers, 2023). Plasma collection could be particularly critical in systems that rely on voluntary unpaid plasma collection; donor management is experiencing and will experience important challenges due to demographic changes and, in some countries, competing commercial and remunerated activities (De Kort, 2010). For example, in Italy, by law (Ministero della Salute, 2015) only healthy individuals aged between 18 and 65 years may donate and, in rare cases, this age can be increased to 67–68 years, based on the physician’s discretion. According to demographic projections by the Italian National Institute of Statistics (ISTAT), the resident Italian population will significantly decline, and according to estimates from ISTAT (ISTAT, 2018), in 2030 the average age of the Italian population will be > 50 years. In addition, around one-third of the total population will be ≥ 65 years of age. Consequently, the population potentially eligible for donation will decrease, whereas the number of those potentially requiring PDMPs will increase.

Increasing plasma collection may not be decisive because companies will suffer from the impact of the last liter economics. Shortage particularly affects the sustainability of IGs because plasma collection costs result in decreasing marginal revenue (last liter economics). Notwithstanding, actions have been taken by pharmaceutical companies, such as increasing the number of centers, improving production efficiency, and improving accessibility of plasma collection centers.

Some initiatives have also been implemented at the European level (Brand et al., 2021), regarding plasma collection, such as the “Supply” Program, aimed at identifying the best practice, formulating recommendations to disseminate and harmonize it, ensuring an increase in plasma collection by transfusion services, and formulating a proposal for a common European policy capable of mitigating the effects of the decrease in plasma availability with the consequent increase in dependence on non-European countries.

The third action is to enhance the technical efficiency of the production process, thus increasing the yield per liter of the collected plasma. In the last 10 years, there has been a significant commitment by pharmaceutical companies to improve production efficiency and increase fractioning capacity, also benefiting the public system (De Angelis et al., 2019; Farrugia et al., 2019). Additionally, moving from a monopolistic to a competitive tender system for the production of PDMPs on behalf of the INHS led to an increase in the yield per liter of fractionated plasma (De Angelis and Breda, 2019). However, some publications have highlighted that there remains room for greater efficiency, for example, standardizing the production costs of PDMPs obtained from national plasma, which exhibits huge variability among blood establishments, and improving the efficiency of plasma collection (Grazzini et al., 2013).

If the shortage is diffused and generated by a general increase in the cost of raw materials, as is the case for plasma, and if this cost increase is not sustainable and generates losses, the solution could be to increase the price of PDMPs, especially IG. A price increase reduces the losses of the last liter of plasma collected.

The shortage could also be motivated, at the local level, by lower prices compared to those of other countries or unfavorable regulations that align prices with other countries but lower actual revenues (e.g., clawback systems). Since the cost of plasma collection may no longer be sustainable, companies may allocate scarce products to the most remunerative markets once prioritized unmet needs have been satisfied, and direct or indirect increases in prices would make more attractive markets where low prices cannot be afforded by pharmaceutical companies.

An increase in the price of IG was awarded to pharmaceutical companies in Germany and the Nordic Countries. In Italy, a price increase could be granted if the cost of raw materials increases (AIFA, 2020) and it has been recognized by AIFA.

Regarding clawbacks/paybacks, there is evidence that this policy has a negative effect on PDMP availability. In Italy, there is a dual payback regulation that is applied to PDMPs procured in the open market and not applied to PDMPs produced on behalf of the INHS. Extending the payback exemption to all PDMPs would make Italy more attractive to companies that produce commercial PDMPs and thereby reduce shortages in Italy.

The last policy which is worth mentioning is improving allocative efficiency from the supply and demand side, that is optimizing the use of plasma reallocating it from clinical use to PDMPs.

The increase in plasma collection can be particularly addressed to plasmapheresis, which ensures a greater frequency of collection. According to Italian law, for example, plasmapheresis may be performed up to 12 times per year, unlike the donation of whole blood or red blood cells, which may be performed up to four times per year. In Italy, often plasma donation is not deemed to be as important as red blood cell donation. Conversely, it is necessary to update this outdated vision and mention the current standard of care in transfusion medicine, which is represented by Patient Blood Management, defined as “a patient-centered, systematic, evidence-based approach to improve patient outcomes by managing and preserving a patient’s own blood, while promoting patient safety and empowerment” (Shander et al., 2022).

The approach involves timely and multidisciplinary application of evidence-based medical and surgical concepts aimed at (1) detecting early and treating anemia appropriately, (2) minimizing surgical, procedural, and iatrogenic blood loss and managing coagulopathic bleeding in the care setting, and (3) supporting the patient when treated with the most appropriate therapy. This systematic and multimodal approach has been shown to be effective in reducing the number and the volume of red blood cell transfusions, thereby enabling the successful execution of complex interventions without the use of blood components (Shander et al., 2016; Costanzo et al., 2020). In addition, it has also led to a decrease in negative outcomes, such as mortality, morbidity, healthcare-associated infections, and costs (Leahy et al., 2017). The program has also been demonstrated to reduce the risk of medico-legal disputes arising from transfusion administration (Bolcato et al., 2020). Accumulating scientific evidence about the results of the program shows that several red blood cell transfusions may be avoided making them less necessary, rather than becoming the last expendable resource at the end of a complex process. Conversely, there are no similar possibilities or alternative strategies in case of need for life-saving blood products. Therefore, institutions and healthcare stakeholders should increasingly encourage the shift towards the “yellow donation” (plasma donation). This approach should be cultivated from a social perspective by educating and training donors to understand the current needs in the transfusion world. Patient Blood Management could play a central role in increasing the production of PDMPs, decreasing the clinical use of plasma, and consequently increasing its contribution to plasma derivation. Currently (Liumbruno et al., 2009; Roback et al., 2010; Green et al., 2018), the indications for the clinical use of plasma are extremely limited; they are associated with rare situations, such as hemolytic purpura or other microangiopathies, and the correction of coagulation deficiencies when specific coagulation factors are not available. In the latter case, it should be highlighted that several coagulation factors are marketed that are highly effective, thereby reducing the need for plasma administration (which may result in several transfusion risks and issues). Consequently, the indications for plasma transfusion are limited. Despite this, at least in Italy, it is used in a massive and inappropriate manner to increase the circulating fluid mass or reverse the effects of anticoagulants (Desborough and Stanworth, 2013; Chai-Adisaksopha et al., 2016). This ineffective use of plasma involves its subtraction from plasmapheresis activities, and shows the need for a broad educational intervention on the transfusion appropriateness of this blood component. A virtuous experience reported in the literature shows the results of a “zero tolerance” policy on the use of this blood component (Beverina et al., 2019). This led to a >70% reduction in the use of plasma compared to the previous 5 years thereby facilitating a substantial increase in the volume of plasma supplied for industrial processing (Beverina et al., 2019).

## Conclusion

The shortage of PDMPs, particularly IGs, is an important public health issue. Despite the pandemic exacerbating this problem and having a considerable negative impact on plasma collection volumes and costs, it is likely that the shortage will persist in the future. The increasing costs of plasma and the last liter economics could make it unsustainable for pharmaceutical companies to produce more PDMPs.

Ethical considerations and market regulations should be the drivers of any policy aimed at addressing an imbalance between the demand and supply of PDMPs and the consequent PDMP shortage. This would start from a recognition that self-sufficiency very unlikely will fully satisfy the demand for PDMP and should be integrated with commercial PDMP.

A broader vision of the causes of this shortage could provide solutions in the short- and long-term. An appropriate planning is crucial, considering that the supply is quite rigid and does not immediately respond to a demand increase.

Improving technical and allocative efficiency and providing incentives to all stakeholders in the PDMP market should be pursued. Technical efficiency refers to an increase in the ratio between output (PDMPs) and input (plasma). Allocative efficiency refers to both the production aspect (e.g., reducing the role of plasma in clinical practice and relying more on plasmapheresis) and the demand aspect (e.g., making the demand more appropriate and addressing the use of PMPDs to effectively prioritize needs). Incentives to all stakeholders may include indirect ones for donors and higher prices and/or better regulation (e.g., no clawbacks/paybacks) for PDMPs to compensate for the rising plasma costs and the decreasing marginal revenue generated by an additional unit of plasma per liter (last liter economics). There are several factors to be considered in addressing this public health challenge, which should inspire concerted actions based on a broad vision that prompts all stakeholders, both public and private, to adopt decisive action.
